# Antithrombotic Therapy in Cardiac Embolism

**DOI:** 10.2174/157340310791658749

**Published:** 2010-08

**Authors:** Álvaro Cervera, Ángel Chamorro

**Affiliations:** Comprehensive Stroke Center, Hospital Clínic; Institut d’Investigacions Biomèdiques August Pi i Sunyer (IDIBAPS); Barcelona, Spain

**Keywords:** Anticoagulation, heparin, oral anticoagulants, prevention, inflammation.

## Abstract

Anticoagulation is indicated in most cardioembolic ischemic strokes for secondary prevention. In many cardiac conditions, anticoagulation is also indication for primary stroke prevention, mainly when associated to vascular risk factors. Anticoagulation should be started as soon as possible, as it is safe even in moderate acute strokes. The efficacy of early anticoagulation after cardioembolic stroke in relation to outcome has not been assessed adequately, but there is evidence from animal models and clinical studies that anticoagulation with unfractionated heparin is associated with a better outcome mediated in part by its anti-inflammatory properties.

## ANTICOAGULATION IN CARDIOEMBOLIC STROKE PREVENTION

Oral anticoagulation (OAC) is the treatment of choice for secondary prevention after a cardioembolic stroke [1, 2]. Warfarin is the commonest OAC used worldwide, although acenocumarol, phenprocoumon or anisidione are frequently prescribed in many countries. The mechanisms of action of these OAC are comparable, as they inhibit the vitamin K-dependent post-translational carboxylation of glutamate residues on the N-terminal regions of coagulation factors II, VII, IX, and X by inhibiting the conversion of vitamin 2,3 epoxide to reduced vitamin K [3]. Although the benefits of OAC are supported by a high degree of evidence for stroke prevention in cardioembolic entities, such as atrial fibrillation [4], they have a narrow therapeutic index, numerous drug and dietary interactions, and a significant risk of serious bleeding, including hemorrhagic stroke [5]. 

### Atrial Fibrillation

Atrial fibrillation (AF) is the most frequent cardiac condition associated to the risk of ischemic stroke, although it is only weakly associated with transient ischemic attack (TIA) [6]. The incidence of ischemic stroke among patients with AF not treated with antithrombotic agents averages 4.5% per year, and it may be as high as 13% per year in certain high-risk groups. Overall, AF increases the risk of stroke fourfold to fivefold across all age groups [7]. Patient-level meta-analyses of the efficacy of antithrombotic therapies in AF from pooled data of randomized trials showed that adjusted-dose oral anticoagulation (target International Normalized Ratio (INR) 2.5; range, 2.0-3.0) resulted in a relative risk reduction of 68% (95%CI 50%-70%) compared to no antithrombotic therapy [7]. Oral anticoagulation (INR 2.0–3.0) reduces the risk of recurrent stroke in patients with non-valvular AF, regardless of the type (permanent, chronic or paroxysmal) [8]. Aspirin resulted in a relative risk reduction of 21% (95%CI 0%-38%) compared to no antithrombotic therapy [9], and adjusted-dose oral anticoagulation resulted in a relative risk reduction of 52% (95%CI 37%-63%) compared to aspirin, respectively [10]. 

In primary prevention studies OAC lowered the mortality rate by 33% (95%CI 9%-51%), and the combined outcome of stroke, systemic embolism, and death by 48% (95%CI 34%-60%) [11]. In these studies, the reported annual incidence of major bleeding and intracranial hemorrhage was 1.3% and 0.3% in anticoagulated patients, compared to 1% and 0.1% in control patients. The risk of intracranial hemorrhage is significantly increased at INR values >4.0, with increasing age, and in patients with a history of stroke [12].

From the available information it is clear that oral anticoagulation is more efficacious and more risky than aspirin to prevent first stroke in patients with AF [2]. Despite the encouraging results of OAC in AF, this treatment is underutilized in clinical practice as more than one third of eligible patients in primary care practice are not receiving it [13], and subtherapeutic INR are encountered in 45% of patients taking OAC [14].

Several risk stratification schemes have been developed in order to maximize the benefits of the antithrombotic treatment to prevent the risk of first stroke in individual patients (Table **1**). Primary prevention patients whose stroke risk exceeds 4 per 100 patient-years on aspirin benefit from oral anticoagulation [11]. Stroke prone patients are reliably identified by a CHADS(2) score > 3, and they have an average risk of 5.5 strokes per 100 patient-years on aspirin [15]. High risk primary prevention patients are less well identified with the other schemes described in the table. Yet, all schemes are equally sensitive to detect low-risk patients whose stroke rate is 1.4 or lower per 100 patient-years of aspirin. Oral anticoagulation is more effective in patients with AF who have one or more risk factors, such as previous systemic embolism, age over 75 years, high blood pressure or poor left ventricular function [16].

There has been some concern about the risk/benefit of oral anticoagulation in elderly patients, because of a greater risk of hemorrhagic complications in this group of patients. However, the WASPO (Warfarin vs. Aspirin for Stroke Prevention in Octogenarians) [17] and BAFTA (Birmingham Atrial Fibrillation Treatment of the Aged) [18] trials have shown that OAC is safe and effective in older individuals. Therefore, there is no justification to avoid anticoagulation in very old individuals with AF, unless there is a clear contraindication. 

Other approach that has been tested in the prevention of vascular events in patients with AF is the combination of aspirin and clopidogrel. The ACTIVE W (Atrial fibrillation Clopidogrel Trial with Irbesartan for prevention of Vascular Events) study found that the combination of aspirin and clopidogrel was less effective than warfarin and had a similar bleeding rate [19]. In the ACTIVE A trial, 7554 patients with AF who were considered unsuitable to receive vitamin-K antagonist therapy were randomized to received clopidogrel (75 mg/day) or placebo added to aspirin [20]. The addition of clopidogrel to aspirin reduced the rate of major vascular events from 7.6% per year to 6.8%, primarily due to a reduction in the rate of stroke [20]. However, the rate of major hemorrhage increased from 1.3% to 2.0% per year. The trial suggests that there is an important role of abnormal platelet activation in the pathogenesis of stroke in atrial fibrillation, and that antiplatelet combination therapy could be beneficial in patients considered to be at risk if treated with vitamin-K antagonist. One disabling of fatal stroke would be prevented for every approximately 200 patients treated for 1 year [21]. However, the composite of the primary outcome and major hemorrhages between groups was similar, with 968 events in the clopidogrel and aspirin group versus 966 events in the aspirin-only group [22]. One of the main problems of the study is that there was not a clear definition of which groups of patients are unsuitable to receive oral anticoagulation therapy. In the light of the results of these trials, OAC should be the preferred and recommended therapy for the prevention of stroke in patients with AF at a high risk for vascular events [23]. Moreover, only 23% of the patients recruited in the trial had a verified cause of increased bleeding risk that made them really unsuitable for OAC therapy. A major lesson of this observation is the urgent need to implement in future clinical trials more refined ways to identify patient and clinical preferences for health states and treatments once AF is identified [23]. Underutilization of OAC therapy in AF is more consistent with physician rather than patient values [24].

It has been argued that an individualized stroke prediction, particularly in low risk patients, could improve the risk benefit of anticoagulation in AF. In some studies [25, 26] prothrombin activation fragment 1.2 (F1.2) and thrombin-antithrombin III complexes (TAT), markers of thrombin generation, fibrinopeptide A (FPA), reflecting thrombin activity, D-dimer, a breakdown product of fibrin, and B-thromboglobulin and platelet factor 4, were found elevated in plasma in patients with AF. Levels may be higher in patients with spontaneous echo contrast [27]. Higher hematocrit, plasma viscosity, erythrocyte sedimentation rate, fibrinogen, and membrane-bound and soluble P-selectin, were also found independently associated with spontaneous echo contrast in patients with AF [28]. Persistently elevated levels of D-dimer and tissue plasminogen activator antigen during oral anticoagulant therapy have been identified in individuals at risk for cardiovascular events [29]. C-reactive protein was found increased in patients with AF compared with controls [30], and it was recently been suggested as an independent predictor of future development of AF [31]. Nonetheless, biomarkers are not widely used in clinical practice to identify stroke prone AF.

There are other alternatives to OAC that have been explored for stroke prevention in AF patients, such as indobufen, a reversible inhibitor of platelet cyclooxygenase activity. The SIFA trial was a prospective, randomized, open study involving a total of 916 patients with non-valvular AF and a recent cerebral ischemic episode. Patients received either indobufen (100 or 200 mg BID) or warfarin (INR 2.0 to 3.5) for 12 months. The combined incidence of nonfatal stroke (including intracerebral bleeding), pulmonary or systemic embolism, nonfatal myocardial infarction, and vascular death was not significantly different between the two treatment groups [32]. However, the limited power of the study did not exclude the existence of substantial differences between the two treatments. 

Ximelagatran is an oral direct thrombin inhibitor that inhibits the conversion of fibrinogen to insoluble fibrin by thrombin which has also been explored in patients with AF. The drug binds only to the active site of thrombin and does it reversibly, inhibiting not only free thrombin but also clotbound thrombin. In the SPORTIF III trial [33], patients with AF were randomized to treatment with a fixed dose of ximelagatran (36 mg twice daily) or warfarin dose-adjusted (target INR 2.0 to 3.0). Primary events occurred in 2.3% of patients taking warfarin and in 1.6% in the ximelagatran group (p=0.1). The rates of combined minor and major hemorrhages were lower with ximelagatran (29.8% vs. 25%; relative risk reduction 14%; p=0.007) [33]. The risk of intracranial hemorrhage was 0.19% per year for warfarin and 0.11% per year in ximelagatran, and the annual rates of ischemic strokes were 1.46% and 1.37%, respectively. Major bleeding occurred at an annual rate of 2.5% in the warfarin-treated group, and 1.9% in the ximelagatran-treated group, a non significant difference. The majority of ischemic strokes were non cardioembolic in origin, typically lacunar or large-artery atherosclerosis-related strokes. However, in 6.1% of patients receiving ximelagatran there was an increase in alanine aminotransferase greater than 3 times the upper limit of normal. In the SPORTIF V trial [34], (a double-blind trial involving relatively high-risk patients with non valvular AF), ximelagatran was not inferior to well-controlled warfarin within the prespecified margin of 2.0% per year for prevention of stroke and systemic embolism. However, 3 deaths with liver failure were reported in the trials, and it was estimated 1 death from hepatic failure among 2300 patients treated [35]. In data presented to the Food and Drug Administration (FDA) on all patients receiving long-term ximelagatran, an increase in alanine aminotransferase >3 x normal occurred in 7.9% of patients compared with 1.2% of patients receiving comparator therapy, leading the FDA to deny approval of ximelagatran because of concerns about hepatotoxicity [36]. Later on the sponsor officially notified the Committee for Medicinal products for Human Use that it whished to withdraw its application for a marketing authorization for ximelagatran for the prevention of stroke associated with AF.

Dabigatran is a potent, direct, competitive inhibitor of thrombin that, like ximelagatran, does not require regular monitoring [37]. In the RE-LY trial (Randomized Evaluation of Long-Term Anticoagulation Therapy) two fixed doses of dabigatran (110 mg or 150 mg, twice daily) administered in a blinded manner were compared to open-label use of warfarin in 18,113 patients with AF [38]. The primary outcome measure was stroke or systemic embolism, and the primary safety outcome was major hemorrhage. Stroke or systemic embolism occurred in 1.53% per year in patients receiving 110 mg of dabigatran, 1.11% per year with 150 mg dabigatran, and 1.69% per year in patients receiving warfarin, with a median duration of follow-up of 2.0 years [38]. Both doses were non-inferior to warfarin, and the 150 mg dose was shown to be superior to warfarin (RR 0.66, 95%CI 0.53 to 0.82). Hemorrhagic stroke happened in 0.38% per year with warfarin, 0.12% per year with 110 mg dabigatran, and 0.10% per year with 150 mg dabigatran. Only major gastrointestinal bleeding was more frequent in patients taking 150 mg dabigatran in comparison to warfarin. In this study, there were no significant increases in liver enzymes with dabigatran [38]. The only adverse event that was more frequent with dabigatran was dyspepsia. The conclusion of this trial was that both doses of dabigatran were non inferior to warfarin in the prevention of stroke or systemic embolism. Moreover, the dose of 150 mg was superior to warfarin for embolic prevention, and the dose of 110 mg produced less hemorrhagic events. Therefore, the authors suggested that the dose of dabigatran could potentially be tailored to take into consideration the risk characteristics of a specific patient [38]. Nevertheless, it has to be taken in consideration that the number of patients needed to be treated with dabigatran at a dose of 150 mg to prevent one non hemorrhagic stroke, in comparison to warfarin, is approximately 357 [39]. For this reason and due to a greater risk of non hemorrhagic side effects and a twice-daily dosing, some authors think that switching to dabigatran would not be of great value in patients on warfarin with a good INR control [39]. 

### Pacemakers

Pacemakers are needed to treat many cardiac conditions, but its presence may obscure the diagnosis of important concomitants factors such as AF. Indeed, many patients with pacemakers develop AF, and some patients with AF have concomitant sinus node dysfunction, thus requiring the use of pacemakers [40]. The lack of diagnose of AF may lead to the omission of appropriate treatment with OAC. Thus, patients with AF after pacemaker implantation may have a 70% higher relative risk of stroke than patients without AF, even after adjustment for important clinical predictors [41]. Patients on pacemakers for sinus node dysfunction had an actuarial incidence of stroke of 3% at one year, and 5% at five years, and 13% at 10 years [42]. Pacemakers have different modes of programming and stimulation, and the incidence of AF and embolism may differ accordingly. The MOST study compared ventricular rate-modulated pacing with dual-chamber rate-modulated pacing in 2,010 patients followed for five years and whose sinus node dysfunction required permanent pacing for bradycardia [43]. The incidence of stroke in the first year after implant was only 2.2% (95% CI 1.6 to 2.9), most likely because of the high use of antithrombotic agents, particularly in patients with AF [43]. Prior stroke, TIA, systemic embolism, age, hypertension, and NYHA functional class before pacemaker implantation, but not mode of pacing, were associated with the risk of stroke [43]. In other studies mortality and incidence of stroke were not reduced by physiologic pacing, although this treatment modality was associated with a significant relative risk reduction in the incidence of AF (18% at 3 years and 20% at 6 years) [44]. 

### Acute Myocardial Infarction

Stroke is a rare but feared complication of acute myocardial infarction (AMI) [45]. The long-term risk of stroke following AMI it is estimated in 1 to 2 % per year. A case-control study showed that stroke secondary to AMI causes a severer neurological deficit, more unfavorable clinical course, and higher mortality than stroke in patients without a recent AMI [46]. The risk of recurrent myocardial infarction, stroke, or death was significantly reduced by OAC compared to aspirin therapy in one study that allocated the antithrombotic regimens within 8 weeks of AMI or unstable angina [47]. Aspirin with medium-intensity OAC was also more effective than aspirin on its own in reduction of subsequent cardiovascular events and death. Therefore, it is recommended that OAC should be taken long term, or for at least 3 months after cardioembolic stroke due to AMI [48].

### Congestive Heart Failure

The incidence of thromboembolism secondary to congestive heart failure (CHF) varies depending on the prospective or retrospective design of the studies, and whether clinical or autopsy data are assessed. Prospective studies of patients with dilated cardiomyopathy have reported a stroke incidence of 1.7 per 100 patient-years [49], while retrospective studies have given an incidence of 3.5 symptomatic events per 100 patient-years [50]. A controlled trial of OAC among patients with CHF has not been performed, although certain groups of patients with CHF have well defined indications for chronic anticoagulation, such as previous thromboembolic event, AF, or the presence of newly formed left ventricular thrombus [51, 52]. Evidence from published reports does not demonstrate convincingly that the benefits of OAC exceed the risks. The SAVE [53] and SOLVD [54] databases have shown that low dose aspirin may be useful in preventing thromboembolism and may be less risky than OAC. In patients with underlying coronary artery disease, aspirin probably confers additional benefit. In the SAVE trial [53], aspirin use significantly reduced the risk of stroke by 56%, and the protective effect of aspirin was most pronounced in patients with a left ventricular ejection fraction < 28%; in this group, aspirin use was associated with a reduction in risk of stroke of 66% (p < 0.001). Similarly, the SOLVD trial [54] showed a beneficial effect of aspirin, especially in women. The use of antiplatelet agents was associated with a 23% reduction in the risk of embolism in men and 53% reduction in women. Aspirin was also associated with a 24% reduction in the risk of sudden death [54]. The V-HeFT studies failed to show any protective effect of anticoagulation [55]. Indeed, V-HeFT II showed that patients receiving anticoagulation had a higher thromboembolic event rate (4.9 per 100 patient-years) than those not receiving anticoagulation (2.1 per 100 patient-years). Because neither the decision to initiate anticoagulation nor the intensity of anticoagulation was controlled, it is likely that patients judged to be at highest embolic risk (e.g., those with AF or known LV thrombus or even patients with mechanical valve replacement) were treated with OAC. 

### Cardiac Procedures

The reported incidence of symptomatic stroke after coronary artery bypass grafting (CABG) is between 0.8% and 5.2% [56]. Coronary bypass surgery without cardiopulmonary bypass (off-pump CABG) is theoretically associated with a lower risk of stroke, given its advantages of no aortic manipulation, no hypothermia, and no use of the cardiopulmonary bypass pump [57]. In a large study with 16,184 patients the incidence of stroke was lower in the off-pump group (2.5%) compared to the conventional CABG group (3.9%) [58]. Embolism has been implicated in the pathophysiology of stroke after on-pump CABG, whereas myocardial stunning and hypoperfusion may be possible mechanisms associated with delayed onset of stroke after off-pump CABG [57]. However, postoperative Diffusion-Weighted Imaging have shown similar rate of ischemic lesions in off-pump as opposed to on-pump CABG [59], suggesting that factors other than the use of the cardiopulmonary bypass are associated with postoperative ischemic lesions. The timely administration of platelet inhibitors and/or per-operative anticoagulation, as well as prevention of hypotensive episodes may be indicated in off-pump CABG as preventive measures against delayed onset of stroke. Yet, further studies are needed to prospectively investigate the potential benefits of pharmaceutical agents in reducing the incidence of stroke after CABG. Acute stroke after percutaneous coronary intervention (PCI), although rare, is associated with high rates of mortality and morbidity [60]. The incidence of stroke and TIA ranges from 0.27% to 0.50% [60, 61]. Appropriate use of antiplatelet agents and anticoagulants during PCI is aimed at improving early clinical outcome, and preventing local complications at the site of intervention which might increase the risk of stroke [62]. 

### Patent Foramen Ovale (PFO)

Patent foramen ovale (PFO) has been identified as a potential cause of stroke, although the association may be age-dependent [63]. Because stroke occurs more frequently in older population, with only 3% of cerebral infarctions occurring in patients younger than 40 years, the number of stroke patients with PFO older than 40 years is much larger than in younger patients [63]. The association of PFO with cryptogenic stroke in older patient populations is conflicting [64]. In the Stroke Prevention: Assessment of Risk in a Community (SPARC) echocardiography study [65], PFO was not a significant independent predictor of stroke (HR 1.46, 95%CI 0.74 to 2.88). The secondary stroke prevention in patients with PFO has been evaluated in several studies. In the PFOASA study young patients (from 18 to 55 years) with cryptogenic stroke within the preceding 3 months were prospectively followed during 4 years of aspirin therapy (300 mg per day)[66]. The risk of recurrent stroke was 2.3% in patients with PFO alone, 15.2% among patients with both PFO and atrial septal aneurysm (ASA), 4.2% among patients with neither of these cardiac abnormalities, and 0% in patients with ASA alone. The PICSS trial evaluated older stroke patients (from 30 to 85) with or without PFO who were randomized to receive aspirin (325 mg) or warfarin (INR 1.7 to 2.2) [67]. Regardless of treatment, the rate of recurrent stroke or death in patients with PFO (9.5%) was not significantly different than in patients without PFO (8.3%). Moreover, larger PFO were associated with a lower, not higher, overall rate of recurrent stroke or death (18.5% with small PFO versus 9.5% with large PFO), and the coexistence of PFO and ASA did not increase stroke risk [67]. Currently, the evidence is insufficient to determine if OAC is superior to aspirin for the prevention of recurrent stroke or death in patients with cryptogenic stroke and PFO, or the value of surgical or endovascular closure. The Randomized Evaluation of Recurrent Stroke Comparing PFO Closure to Established Current Standard of Care Treatment (RESPECT) trial, the CLOSURE I trial, and the CardiaPFO trial are currently comparing medical and percutaneous closure approaches, but large patient enrolment would be necessary due to the low event rate in these patients. 

### Rheumatic Mitral Valve Disease

Recurrent embolism occurs in 30 to 65% of patients with rheumatic mitral valve disease, 60 to 65% during the first year, and most within 6 months. Although never evaluated by randomized clinical trials, there is little doubt that long-term OAC therapy (target INR 2.0-3.0) is effective in reducing the incidence of systemic emboli in these patients [2]. Patients with rheumatic valve disease and AF or with previous systemic embolism should be offered treatment with long-term OAC. Exceptions include pregnant women or the patient at high risk for serious bleeding [68]. In patients with recurrent embolism despite being treated with OAC at a therapeutic INR, is recommended to add aspirin (75-100 mg/d), dipyridamole (400 mg/d), or clopidogrel (75 mg/d) [68]. 

### Mechanical Prosthetic Heart Valves

It is well established that patients with all types of mechanical valves require antithrombotic prophylaxis for stroke prevention [68]. Lack of prophylaxis in patients with St. Jude Medical bileaflet valves is associated to embolism or valve thrombosis in 12% per year with aortic valves, and 22% per year with mitral valves [69]. For patients with St. Jude Medical bileaflet and Medtronic Hall tilting disk mechanical valves in the aortic position, with no AF or enlarged left atrium, a target INR 2.0 - 3.0 is satisfactory [70]. An INR target 2.5-3.5 is satisfactory for tilting disk valves and bileaflet prosthetic valves in the mitral position [71]. In older devices, such as caged ball or caged disk valve, the optimal INR for thromboembolic prevention has be to higher, from 4.0 to 4.9 [71]. The combination of OAC and aspirin may be particularly useful in patients with prosthetic valves who have coronary artery disease or stroke [72]. Available data suggest that neither adjusted-dose unfractionated heparin nor fixed-dose low-molecular weight heparin (LMWH) provide adequate protection in pregnant patients with mechanical heart valves [68].

### Bioprosthetic Heart Valves

In patients with bioprosthetic valves without AF, long-term therapy with aspirin (75-100 mg/d) is recommended [1, 68]. For patients with bioprosthetic valves in the mitral position OAC with a target INR from 2.0 to 3.0 is recommended during the first 3 months after valve insertion [68]. On the other hand, patients with bioprosthetic valves in the aortic position can be given either OAC (INR 2.0-3.0) or aspirin (80-100 mg/d) during the first 3 months after valve insertion [68]. Treatment with OAC has to be longer, from 3 to 12 months, in patients with a history of systemic embolism [68]. 

### Infective Endocarditis

Ischemic stroke is the most common neurological complication of infective endocarditis occurring in approximately 20% of patients. Cerebral emboli are considerably more common in mitral valve endocarditis than in infection of the aortic valve. There is no convincing evidence that prophylactic anticoagulant therapy reduces the incidence of emboli in native valve endocarditis, and it is generally believed that the routine use of anticoagulants is not justified [68]. However, in patients with special indica-tions, such as AF, appropriate anticoagulant therapy should not be withheld [2]. Patients with prosthetic valves are at constant risk of thromboembolism and there are important reasons not to interrupt anticoagulant therapy in this circumstance. Embolic events in prosthetic valve endocarditis may represent dislodged vegetations or true thromboembolism unrelated to valve infection. While the incidence of the latter can be reduced by anticoagulation therapy, there is no evidence that embolic vegetations are controlled with this therapy. In previously undiagnosed patients who present with stroke, and who prove to have cardiac vegetations, it is often challenging to differentiate between infective endocarditis and nonbacterial thrombotic endocarditis (NBTE) [2]. Diagnostic clues such as fever or Roth’s spots (which suggest infective endocarditis) and metastatic tumors (which suggest NBTE) may be absent, and blood cultures may remain negative. Diffusion-weighted imaging (DWI) can provide additional diagnostic clues for patients with NBTE uniformly have multiple, widely distributed, small and large strokes, whereas patients with IE exhibit a host of different stroke patterns [73]. 

### Nonbacterial Thrombotic Endocarditis

Nonbacterial thrombotic endocarditis (NBTE) is reported most commonly in patients with adenocarcinoma, especially mucin-producing carcinomas of the lung or gastrointestinal tract, and lymphoma. The malignancy is usually widespread and cerebral infarction is a late complication, but in rare instances NBTE with cerebral infarction is the presenting sign of cancer. The reported incidence of systemic embolism in NBTE varies widely (14- 91%, average 42%) [74]. NBTE is more common in the aortic and mitral valves, but any valve may be affected. The pathogenesis of NBTE is not fully understood, but the most important predisposing factors appear to be an underlying coagulopathy, edema, degeneration of valvular collagen, and the effects of mucin-producing carcinomas. Treatment of NBTE is directed toward control of the underlying disease, in most instances neoplasia and/or sepsis, and toward treatment of thromboembolism. The most effective agent is heparin, and little benefit has been observed with vitamin K antagonists. Patients with NBTE and systemic or pulmonary emboli should be treated with full-dose unfractionated heparin IV or subcutaneous heparin [68]. Patients with disseminated cancer or debilitating disease with aseptic vegetations should also be treated with full-dose unfractionated heparin. The therapy of NBTE and cerebral intravascular coagulation is based on attempts to treat the underlying cancer and the activated coagulation. Heparin is more effective than OAC in treating the coagulopathy that accompanies cancer and a recent report confirms that low molecular weight heparin is more effective than OAC in preventing recurrent deep venous thrombosis and pulmonary embolism in cancer patients [75]. 

### Libman-Sacks Endocarditis

Valvular involvement is the most frequent form of heart disease in systemic lupus erythematosus (SLE). Involvement includes valve masses also known as Libman-Sacks vegetations, valve thickening, valve regurgitation, and valve stenosis. On transesophageal echocardiography, the prevalence of valvular disease in SLE has been shown to be up to 60–74%. The incidence of ischemic cerebrovascular stroke in patients with SLE is 10–20%; in these patients, the existence of valvular involvement and left heart thrombi was proven in 70–90% of cases [76]. A frequent concomitant appearance of valvulopathy, thromboembolic events (mostly stroke or TIA) and antiphospholipid antibodies has been observed. Ischemic manifestations, previously thought to be due to vasculitis, are usually due to thrombotic or cardioembolic events. Because of the increased incidence of stroke in SLE and the frequent valvulopathy in these patients, prophylactic antiplatelet therapy may be contemplated in all SLE patients. Anticoagulant treatment should be considered independently of echocardiographic results in patients who had cerebrovascular or systemic embolic events with no features of systemic SLE vasculitis [77]. 

### Mitral Annular Calcification and Aortic Valve Sclerosis

Mitral annular calcification (MAC) is characterized by calcium and lipid deposition in the annular fibrosa of the mitral valve, whereas aortic valve (AV) sclerosis results from similar accumulation involving the AV leaflets. MAC and AV sclerosis are associated with atherosclerosis risk factors that can promote left ventricular hypertrophy and left atrial enlargement, each of which has been reported to predict cerebrovascular events. The ACCP recommends long-term OAC in patients with MAC complicated by systemic embolism not documented to be calcific embolism [68]. For patients with repeated embolic events despite anticoagulation therapy, or in whom multiple calcific emboli are recognized, valve replacement should be considered. 

### Mitral Valve Prolapse

The prevalence of mitral valve prolapse (MVP) in community-based studies is low (2.4%), and no more common among young patients with unexplained cerebral embolic events [78]. Nevertheless, it is recommended that patients with MVP and stroke receive antithrombotic therapy if alternative causes of brain ischemia cannot be identified [68]. Long-term aspirin (50-162 mg/d) is recommended for unexplained stroke in patients with MVP, while long-term OAC has only been suggested for MVP patients with recurrent vascular events despite aspirin [68]. 

### Bleeding Risk in Orally Anticoagulated Patients

The risk of major bleeding in patients receiving OAC is 3% per year; and approximately 20% of major bleeding events are fatal [79]. Even at safe anticoagulant levels (INR 2.0 to 3.0) annual rates of major, life threatening, and fatal bleeding are 2%, 1%, and 0.25%, respectively [80]. Every one-point rise in INR increases the risk of major bleeding by 42% [81] and the interval 2.0-2.5 gives the lowest risk of stroke and death in patients with non valvular AF [82]. Concomitant hypertension, prior cerebrovascular accident, gastrointestinal bleeding or anticoagulation-related bleeding, use of aspirin or non steroidal anti-inflammatory drugs, older age, patient reliability, and the interactions of OAC with other medications contribute to the risk of bleeding [83]. 

The most frequent complication of OAC is gastrointestinal bleeding, but intracranial hemorrhage (ICH) is the main cause of fatal bleeding. In a pooled analysis of the first five trials with warfarin in patients with AF the annual rate of OAC-related ICH was 0.3% [7]. OAC-related ICH occurs at a rate of 2 to 9 per 100 000 population/year, an incidence which is 7 to 10 fold higher than in patients not receiving OAC [84]. The incidence of intracranial hemorrhage due to OAC is increasing, probably because of the larger number of elderly patients that receive this treatment, the association with aspirin, or the expanded use of OAC for stroke prevention [85]. 

### Long-term Secondary Stroke Prevention After OAC-Related ICH

Another difficult decision in clinical practice is whether anticoagulants should be restarted and maintained indefinitely in patients with a history of OAC-related ICH and at risk of cardioembolic events. Stroke prevention in this situation needs to balance the risk/benefit of different antithrombotic options and the estimated risk of intracranial bleeding recurrence. To this aim, an important step is to establish the most likely cause of the bleeding. Whereas hypertensive vasculopathy appears to be the most important mechanism for ICH in deep hemispheric regions of the brain, cerebral amyloid angiopathy, may be the most common underlying pathophysiology for lobar ICH. The risk of recurrent hypertensive ICH can be decreased by an adequate control of hypertension [86], whereas cerebral amyloid angiopathy lacks any known treatment. In a prospective study of elderly patients who survived lobar ICH, recurrent ICH occurred in 22% at 2 years [87]. The rate of recurrent ICH in survivors of deep hemispheric ICH was estimated to be 2.1% per patient-year [88]. Therefore, in patients with lobar hemorrhage and major sources of embolism, decision analysis models based on retrospective data suggest that the strategy of “do not anticoagulate” appears robust [88]. Contrarily, the risks and benefits of anticoagulation are more closely balanced when applied to patients with deep hemispheric ICH. In the latter case, OAC might be justified if the estimated risk of ischemic stroke is high.

## IMMEDIATE ANTIAGULATION AFTER ACUTE CARDIOEMBOLIC STROKE

Unfractionated heparin (UFH) is the most widely used anticoagulant in the acute stroke setting. Heparin binds to antithrombin through a unique glucosamine unit that is contained within a pentasaccharide sequence [89].Therefore, it has a potent anticoagulant effect mediated by this binding to antithrombin that converts it from a slow, progressive thrombin inhibitor to a very rapid inhibitor [90]. However, UFH also has important anti-inflammatory properties [91] that could be of relevance in the acute stroke setting, as inflammation, mainly when there is ischemia followed by reperfusion, is an important mechanism of cell injury [92]. Thus, in a rat model of focal ischemia-reperfusion in the brain, an adjusted-dose of UFH was able to reduce the infarct volume by a 46% in comparison to rats treated with vehicle [93]. The reduction of infarct volume was partly mediated by anti-inflammatory mechanisms, as rats treated with UFH had higher plasmatic levels of interleukin-10, higher brain expression of hemeoxygenase-1, and lower endothelial induction of vascular cell adhesion mollecule-1 [93]. Also, in observational clinical studies, patients with acute stroke treated with UFH showed less prominent rise in serum acute phase reactants than patients treated with aspirin [94]. In another study patients treated with UFH had a lower increase in serum vascular cell adhesion molecule-1 in comparison to patients treated with aspirin [95]. These serologic findings of a reduced systemic inflammation in patients treated with UFH were associated with an improved clinical outcome after stroke [94, 95].

### Clinical Evidence of Anticoagulation in Acute Ischemic Stroke

The value of anticoagulation in the acute stroke setting has been assessed in many different clinical trials. These studies have evaluated the efficacy of UFH, low-molecular-weight heparins, or heparinoids. A meta-analysis of all the trials concluded that there is no role for immediate anticoagulation in acute ischemic stroke [96]. Patients with acute ischemic stroke receiving anticoagulants in the first two weeks of onset has 9 less ischemic recurrences per 1000 patients [97]. However, this benefit is counterbalanced by a similar increase in symptomatic intracranial hemorrhages. Therefore, most clinical guidelines do not recommend anticoagulation in the acute phase of ischemic stroke [1]. The lack of evidence for heparin use in acute stroke has also been found in the subgroup analysis of patients with cardioembolic stroke secondary to AF [98].

It has to be noted that the trials included in these analyses had many methodological problems [99]. For example, in the largest study [100] almost one third of patients had the first neuroimaging after the treatment was allocated, raising the possibility of the erroneous inclusion of approximately 500 intracranial hemorrhages [99]. Moreover, UFH was not monitored in this trial and there was a great delay in the start of treatment. In many of these trials anticoagulation was no really immediate, therefore there is no adequate evidence of the utility of immediate anticoagulation in acute stroke [101]. Most of the studies with anticoagulation in the acute stroke setting allowed a 24 to 48 hours delay in the beginning of drug administration from symptom onset. This delay avoids an adequate neuronal protection, as the majority of events responsible of cell death take place in an earlier phase [99]. Thus, one observational study showed that in stroke patients with AF starting anticoagulation with UFH within the first 6 hours of clinical onset was associated with a better clinical outcome, in comparison with patients in whom anticoagulation was delayed [102].

The Rapid Anticoagulation Prevents Ischemic Damage (RAPID) trial was also a randomized trial evaluating immediate anticoagulation in ischemic stroke [103]. RAPID compared the effect of adjusted-dose UFH and aspirin administered in the first 12 hours of a non lacunar ischemic stroke. The mean treatment delay was 6.9 hours. In this trial it was shown that a higher risk of bleeding was associated to excessive anticoagulation [103]. Therefore, it is very relevant the fact that some trials of anticoagulation in the acute phase of stroke omitted anticoagulation monitoring. RAPID was an academic effort to reconcile science and simplicity, but unfortunately, a hopeless recruitment rate led to the premature termination of the study when only 67 patients had been included. Nevertheless, it was able to show a trend toward more effective prevention of stroke recurrence with UFH (0%) than aspirin (8.6%; P=0.09) and without an increment in serious bleeding (8.6% for aspirin, 6.3% for UH; P=0.71) [103].

Later, in the pre-thrombolytic era, a randomized clinical trial compared the effects of UFH and placebo in the first 3 hours after stroke onset [104]. The trial included 418 patients with an ischemic non lacunar stroke. In the group treated with UFH there was a better outcome at 3 months (self-independent patients, 38.9% vs. 28.6%), fewer deaths (16.8% vs. 21.9%), and more symptomatic brain hemorrhages (6.2% vs. 1.4%), and more major extracerebral bleedings (2.9% vs. 1.4%) [104]. Therefore, this is the first randomized trial to show that UFH administered in the first 3 hours after a non lacunar ischemic stroke is effective in reducing dependence when compared to placebo [104]. Although thrombolytic treatment with alteplase was known to be effective at that time, this treatment was not approved for clinical use in Europe yet. But it is important to keep in mind that the level of evidence for the efficacy of UFH in the acute stroke setting is reasonable. However, as the meta-analysis will always include the larger trials with the above mentioned methodological problems, is highly improbable that UFH will have a role in the acute stroke treatment in the near future. 

### When to Start Anticoagulation After a Cardioembolic Stroke for Secondary Prevention?

Delaying anticoagulation in stroke secondary to AF was advocated years ago to avoid early hemorrhagic transformation. However, from observational studies we know that carefully monitored UFH is safe, even in patients with large infarcts attributable to AF [105]. Thus, hemorrhagic conversion and symptomatic hemorrhage in embolic stroke patients treated with UFH were not related to admission clinical severity or infarct size. The only parameter that was independently associated to hemorrhagic complications was an excessive prolongation of the activated partial thromboplastin time [105]. 

The urgency of full anticoagulation is mainly justified in light of the molecular mechanisms of brain ischemia [106]. The delay of treatment for 24-48 hours is not very important if our primary aim is to decrease the risk of early stroke recurrence. However, treatment delay may be of vital relevance if we aim primarily to improve functional outcome and reduce mortality. 

Stroke secondary to AF has raised special interest because of higher risk of early stroke recurrence, although more recent information opposes this concept [101]. Therefore, anticoagulation for early stroke prevention can be delayed for some days with no significant relevance. Rather, the central issue for immediate anticoagulation in stroke could be defined as the tissue factor (TF) link [101]. TF is the primary cellular initiator of the coagulation cascade *in vivo*, and behaves as a hemostatic envelope diffusely expressed in the adventitia of cerebral vessels. TF expression is also prominent in the human cortex [107]. Accordingly, any acute embolic stroke is a TF-mediated prothrombotic state that could theoretically be opposed with adequate anticoagulation [101]. As UFH achieves faster anticoagulation levels than any other anticoagulant regimen, this therapy has been recommended for acute stroke patients if there are not contraindications [101]. For the efficacy of this treatment it is necessary to calibrate the activated partial thromboplastin time ratios to their corresponding heparin levels (0.3 to 0.5 U/mL), careful monitoring, and frequent dose adjustments [103]. 

### Risk/Benefits of Early Anticoagulation in Patients with OAC-Related ICH

A therapeutic dilemma arises when a patient who requires full-dose anticoagulation for high risk of thromboembolism is admitted with OAC-related ICH. Bertram and col. retrospectively studied 15 patients with serious cardiac conditions and ICH which occurred under anticoagulation [108]. In all instances, INR normalization was attempted as early as possible and received full-dose intravenous or low-dose subcutaneous heparin. All patients that achieved a 1.5- to 2-fold elevation in partial thromboplastin time after normalization of the INR were discharged without complication. Alternatively, patients with only incomplete correction of the INR experienced relevant rebleeding within 3 days, and 3 of 7 of the patients with normalized INR and without significant PTT elevation developed severe cerebral embolism. Rebleeding or expansion of the hemorrhage was associated with an INR > 1.5 after previous INR normalization[108]. Small retrospective studies also showed with few exceptions [109] that none of the patients experienced rebleeding or embolic events after initiation of full dose heparin within 36 to 72 hours of OAC-ICH onset [110]. In one study early resumption of anticoagulation did not cause intracranial rebleeding even in patients that undergo early surgery of the hematoma [111].

A retrospective series of 141 patients at the Mayo Clinic showed that the risk of having an ischemic stroke after discontinuation of OAC within 30 days of the ICH was less than 5%, although the mortality was 48% [112]. All embolic events occurred within the first 5 days after warfarin discontinuation. Of the 34 patients in whom anticoagulation therapy was restarted by day 14, none had recurrence of ICH during hospitalization. Tinker and Tarhan also observed in 159 patients with mechanical heart valves undergoing elective surgery that none of the patients had in-hospital thromboembolic complications after discontinuing warfarin therapy [113]. Based on this small data base, the early initiation of full-dose anticoagulation in patients with OAC-ICH cannot be recommended (or opposed). While early initiation of full-dose anticoagulation was not followed in these studies by a clear reduction of the risk of embolism, the low statistical power of the studies cannot rule out significant differences. Moreover, few patients had the biological effects of heparin adequately monitored in these studies, which is a crucial step for heparin safety [108]. Avoiding heparin for 1 to 2 weeks after the OAC-related ICH yields a complete reduction of rebleeding, but the incidence of thrombin-mediated hematoma growth or edema formation it is not known. 

Weight-adjusted heparin has been used safely in other bleeding conditions of the central nervous system, including hemorrhagic stroke [105, 114] or cerebral venous infarctions [115]. In these diseases, the bleeding respond to mechanisms different from OAC-related ICH but serve to illustrate that heparin does not prevent full recovery of certain brain hematomas. Lacking randomized clinical trials to establish the value of full anticoagulation in patients with OAC-ICH, therapeutic decisions should be consider case by case. Yet, clinicians should be aware that full anticoagulation must not be started before stable blockade of OAC effects. The greater safety of weight-adjusted anticoagulant nomograms, and the importance of strict monitoring of the biological effects of heparin have also to be considered [106]. 

## Figures and Tables

**Table 1:** Stroke Risk Stratifications Schemes in Patients with Non-Valvular Atrial Fibrillation. (BP: Blood Pressure, DM: Diabetes Mellitus, CHF: Congestive Heart Failure, TIA: Transient Ischemic Attack, CAD: Coronary Artery Disease, LV: Left Ventricular Fractional Shortening)

Scheme	Low Risk	Moderate Risk	High Risk
AFI [7]	Not moderate/high risk	Age>65, not high risk	Prior ischemia, High BP, DM
SPAF [116]	Not moderate/high risk	High BP, not high risk	Prior ischemia, female>75 yrs, CHF, LV < 25%, systolic BP > 160
ACCP [11, 117]	Not moderate/high risk	1 of: 65-75 yrs, DM, CAD and not high risk	Prior ischemia, high BP, CHF, >75 yrs, or ≥ 2 moderate risk factors
CHADS2 [15]	SCORE=+1 for CHF, high BP, DM, >75yr, and +2 for prior stroke/TIA		
FRAMINGHAM [118]	SCORE=+6 for prior ischemia, 0 to 4 for BP, +4 for DM, + 0 to 10 for age, 6 for female		
